# Squamous Cell Carcinoma Developing from a Testicular Epidermal Cyst: A Case Report and Literature Review

**DOI:** 10.1155/2019/9014301

**Published:** 2019-03-24

**Authors:** Ryo Kasahara, Ryosuke Tajiri, Kota Kobayashi, Masahiro Yao, Kazuo Kitami

**Affiliations:** ^1^Department of Urology, Fujisawa City Hospital, 2-6-1 Fujisawa, Fujisawa City, Kanagawa Prefecture, Japan; ^2^Department of Urology, Yokohama City University School of Medicine, 3-9 Fukuura, Kanazawa Ward, Yokohama City, Kanagawa Prefecture, Japan; ^3^Department of Pathology, Fujisawa City Hospital, 2-6-1 Fujisawa, Fujisawa City, Kanagawa Prefecture, Japan

## Abstract

A 50-year-old Japanese man with a two-year history of a painless right scrotal mass visited our hospital. Considering laboratory findings and computed tomography, the patient was diagnosed with an uncharacteristic testicular tumor. No metastases were present on radiographic study at the first visit. Emergent high radical orchiectomy was performed, and the tumor was identified as a squamous cell carcinoma (SCC) of a testicular epidermal cyst. He is alive without recurrence or metastasis six months after surgery. Testicular SCC is an extremely rare tumor. This is the third case of testicular SCC associated with an epidermal cyst in English literature.

## 1. Introduction

A large proportion of testicular cancers are seminomas, and most nonseminomas include embryonal carcinomas and yolk sac tumors [1]. We encountered a case of primary squamous cell carcinoma (SCC) of an epidermal cyst in the testis. Testicular SCC is extremely rare, except for metastasis from other primary lesions. Herein, we describe our experience with this case and review the relevant English literature.

## 2. Case Presentation

A 50-year-old Japanese man with a two-year history of a painless right scrotal mass visited our hospital. He was previously healthy and did not take any medications. His scrotal mass was elastic and hard and had no translucency. We palpated normal testis and epididymis in the contralateral scrotum.

**Figure 1:**
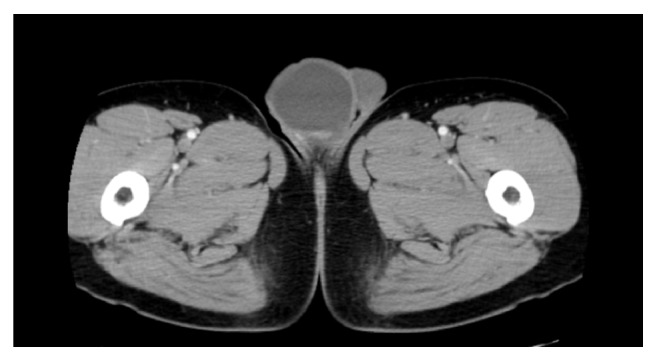
CT revealed a 48 x 48 x 42 mm cystic tumor. The wall of the cystic structure was thickened and enhanced with contrast medium.

We ordered blood laboratory examination and computed tomography (CT). Laboratory data were largely unremarkable and testicular tumor markers were not elevated (alpha-fetoprotein (AFP): 2.9 ng/mL; beta human chorionic gonadotropin (hCG): <0.1 ng/mL). CT revealed a testicular tumor with cystic structure in the right scrotum. The wall of the cystic structure was thickened and enhanced with contrast medium. The tumor size was 48 x 48 x 42 mm (Figure 1). Considering clinical and radiological findings, his scrotum mass was considered to be an uncharacteristic testicular tumor. CT showed no metastasis to lymph nodes or other organs.

Emergent high radical orchiectomy was performed. The operation time was 36 minutes and there was minimal bleeding. The resected tumor was cystic and filled with the brown pus-like fluid. He was discharged from our hospital on the second postoperative day.

**Figure 2:**
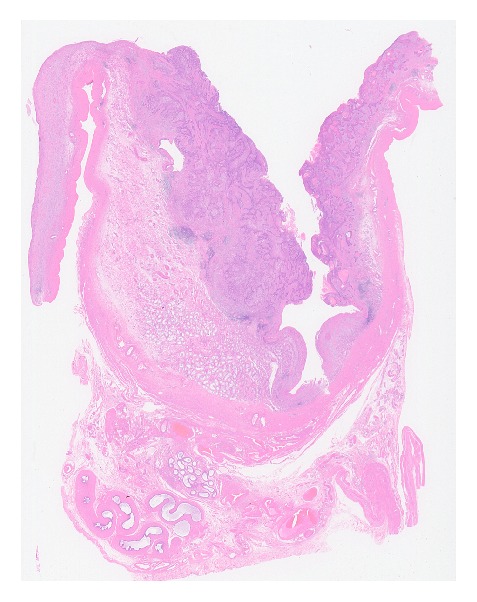
Loupe image of the tumor shows squamous cell carcinoma developing from the cyst in the parenchyma of the testis.

**Figure 3:**
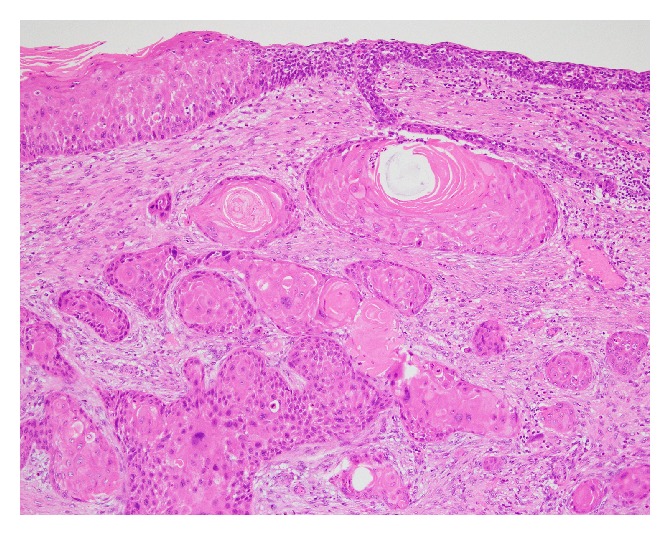
The neoplasm contained a cancer pearl which is consistent with typical squamous cell carcinoma.

The specimen was submitted for pathological examination. Hematoxylin-eosin staining revealed SCC developing from the cyst in the parenchyma of the testis (Figure 2). The neoplasm contained a cancer pearl and was consistent with typical SCC (Figure 3). The cyst did not have cutaneous appendages, bone, or cartilage; therefore, it was considered to be a simple epidermal cyst, not a dermoid cyst or teratoma. Intraepithelial carcinoma was present in the epidermal cyst. As a result, the tumor was considered a primary lesion, not a metastasis. An area of normal testicular parenchyma remained. The final diagnosis was SCC developing from a testicular epidermal cyst.

After discharge, he was followed up with CT and tumor marker (SCC). At six months after operation, SCC was within the normal range (1.1 ng/mL) and CT showed neither recurrence nor metastasis.

## 3. Discussion

Most primary testicular tumors are germ cell tumors, and primary testicular SCCs are very rare, with only three cases reported in the English literature [2–5].

The incidence of secondary (metastatic) testicular tumor has been reported as 0.06%, with origins including the lung, prostate, gastrointestinal tract, skin (melanoma), and kidney [2, 6]. We thought that this low incidence was, for the most part, the result of the blood-testis barrier and the scrotum, which keeps the testes at a low temperature. The blood-testis barrier is one of the tightest junctions in the human body and is composed of four different cells [7]. The scrotum, which works as a radiator, keeps the testes at a low temperature. These functions may make invasion and proliferation of metastatic tumor cells difficult. Furthermore, there are two case reports of testicular metastasis of SCC from the lung [8, 9]. Hence, it is important to determine whether the tumor is a primary tumor and other neoplasms should be excluded.

With regard to the diagnosis of testicular SCCs, it is common to suspect a malignant transformation of a teratoma or from other origins. In this case, the carcinoma developed from an intraepithelial tumor of the cyst, and the cyst did not have cutaneous appendages, bone, or cartilage. CT also revealed no other neoplastic lesions. From the abovementioned information, the tumor was diagnosed as a primary tumor that developed from an epidermal cyst.

Up to now, three cases of primary SCCs of the testis have been reported in the English literature [3–5]. Bryan et al. reported a case of a testicular tumor derived from the malignant transformation of an epidermal cyst [3]. Shih et al. described a case involving testicular SCC that arose from the malignant transformation of a hydrocele [4]. Kim et al. reported the case of a testicular tumor derived from the malignant transformation of an epidermal cyst [5].

Shah et al. collected 141 cases of testicular epidermal cysts [10], with patients aged from 3 to 77 years old. Ethnic grouping analysis revealed that testicular epidermal cysts were more common in white patients (92 patients) and 25 Japanese patients were included. Epidermal cysts of the testes account for less than 1% of all testicular tumors, are frequently benign, and have good prognoses. However, several cases demonstrated loss of heterozygosity for a certain chromosomal loci, supporting the possibility of neoplastic potential [10].

In general, squamous cell carcinogenesis has been thought to be related to chronic irritation and inflammation. However, with regard to testicular SCCs alone, it is unclear whether such mechanisms exist.

Due to its rarity, there are no guidelines or prognostic data available. However, as previously mentioned, epidermal cysts have the potential for malignant transformation. All SCC cases developing from epidermal cysts have been reported in patients over 50 years old [3, 5]. Therefore, there is the possibility that epidermal cysts in older patients may be complicated with neoplasm. Hence, it may be permissible to follow up older patients with epidermal cysts using SCC tumor marker and CT for several years.

We reported the third case of testicular SCC associated with an epidermal cyst in English literature.
